# A Blended Physiotherapy Intervention for Persons With Hemophilic Arthropathy: Development Study

**DOI:** 10.2196/16631

**Published:** 2020-06-19

**Authors:** Merel A Timmer, Corelien J J Kloek, Piet de Kleijn, Isolde A R Kuijlaars, Roger E G Schutgens, Cindy Veenhof, Martijn F Pisters

**Affiliations:** 1 van Creveldkliniek University Medical Center Utrecht Utrecht Netherlands; 2 Research Group Innovation of Human Movement Care University of Applied Sciences Utrecht Utrecht Netherlands; 3 Physical Therapy Research, Department of Rehabilitation, Physical Therapy Science and Sport, Brain Center Rudolf Magnus University Medical Center Utrecht Utrecht Netherlands

**Keywords:** hemophilia, physiotherapy, exercise, eHealth, blended care, mobile phone

## Abstract

**Background:**

Joint bleeds are the hallmark of hemophilia, leading to a painful arthritic condition called as hemophilic arthropathy (HA). Exercise programs are frequently used to improve the physical functioning in persons with HA. As hemophilia is a rare disease, there are not many physiotherapists who are experienced in the field of hemophilia, and regular physiotherapy sessions with an experienced physiotherapist in the field of hemophilia are not feasible for persons with HA. Blended care is an innovative intervention that can support persons with HA at home to perform the advised physical activities and exercises and provide self-management information.

**Objective:**

The aim of this study was to develop a blended physiotherapy intervention for persons with HA.

**Methods:**

The blended physiotherapy intervention, namely, e-Exercise HA was developed by cocreation with physiotherapists, persons with HA, software developers, and researchers. The content of e-Exercise HA was compiled using the first 3 steps of the Center for eHealth Research roadmap model (ie, contextual inquiry, value specification, and design), including people with experience in the development of previous blended physiotherapy interventions, a literature search, and focus groups.

**Results:**

A 12-week blended intervention was developed, integrating face-to-face physiotherapy sessions with a web-based app. The intervention consists of information modules for persons with HA and information modules for physiotherapists, a graded activity program using a self-chosen activity, and personalized video-supported exercises. The information modules consist of text blocks, videos, and reflective questions. The patients can receive pop-ups as reminders and give feedback on the performance of the prescribed activities.

**Conclusions:**

In this study, we developed a blended physiotherapy intervention for persons with HA, which consists of information modules, a graded activity program, and personalized video-supported exercises.

## Introduction

Hemophilia is an X-linked congenital disorder that impairs the body’s ability to make blood clots [1]. It is a rare disease, with a prevalence of 1 in 10,000 persons. Hemophilia is characterized by prolonged bleeding after injuries, easy bruising, and an increased risk of bleeding in the joints and muscles. When untreated, persons with severe hemophilia (<1% blood clotting factors) experience spontaneous bleeding, whereas persons with mild hemophilia (>5% blood clotting factors) experience bleeding only after a trauma [1]. Despite the introduction of prophylactic clotting factor replacement therapy in developed countries in the 1970s, approximately 2 joint bleeds per year are still observed in persons with severe hemophilia receiving prophylactic treatment [2]. The most affected body parts in persons with hemophilia are the ankle joints (42%), knees (20%), and elbows (20%) [3].

Recurrent bleeding in the joints eventually causes hemophilic arthropathy (HA). The pathogenesis of HA has certain features in common with inflammatory joint diseases, but HA is predominantly a degenerative joint disease, which is comparable to osteoarthritis (OA) [4]. The clinical symptoms of pain and the limited range of motion of the joints and atrophy of the surrounding muscles in HA are similar to those reported in OA. Furthermore, people with HA and OA experience limitations in their activities and participation and they are less active than their healthy peers [5].

A wide variety of exercise programs have been developed for persons with hemophilia [6]. However, since these interventions have shown mixed success, a preferred exercise intervention for persons with HA is still undecided. Moreover, regular physiotherapy is not feasible for many persons with hemophilia because physiotherapy treatment is often not covered by health insurance and physiotherapists experienced in hemophilia care are scarce. Since the symptoms and pathogenesis of HA are similar to those of OA, it can be hypothesized that increasing physical activity in daily life has the potential to improve the physical functioning in persons with HA, as previously shown in persons with OA [7]. To our knowledge, no interventions that target the daily movement behavior of persons with hemophilia have been described to date.

Medical apps in smartphones are an upcoming phenomenon in health care and they offer unique possibilities for providing information, supporting behavioral change, and encouraging self-control. Integrating a medical app in smartphones with regular face-to-face physiotherapy sessions can support the participants in performing the advised physical activity behavior and prescribed home exercises and support self-management beyond the walls of the physiotherapy practice. Previously developed electronic health (eHealth) apps for persons with hemophilia include videoconferencing apps and apps that report bleeding events and the use of clotting factor replacement therapy [8,9]. An eHealth intervention directed at changing the movement behavior and improving physical functioning is not yet available for persons with hemophilia.

Recently, a blended exercise program “e-Exercise” was developed for persons with OA, integrating face-to-face physiotherapy with a smartphone app [10]. This program consists of information modules on increasing self-management, personalized exercises, and a behavioral graded activity approach. A clustered randomized controlled trial showed that the effectiveness of e-Exercise in patients with OA was comparable to that of the usual physiotherapy in these patients [11]. Moreover, patients with OA in the e-Exercise group needed only 5 face-to-face sessions, whereas patients in the usual physiotherapy group needed 12 face-to-face sessions.

A blended physiotherapy intervention is expected to be beneficial for persons with HA because it has the potential to support behavioral change and it creates the opportunity to treat patients who have limited access to specialized physiotherapy. Furthermore, it could be used to support physiotherapists in primary health care, who are less familiar and not experienced in treating persons with HA. We hypothesized that certain aspects of the previously developed e-Exercise OA intervention could be suitable for persons with HA as well, given the previously mentioned similarities between the two disorders. However, significant changes might be needed in the intervention for HA because of the differences in the congenital character of hemophilia, possible fear of bleeding, involvement of multiple joints, and involvement of other joints (eg, ankles). The aim of this study was to develop a blended physiotherapy intervention for persons with HA. The proposed program was developed upon the existing e-Exercise intervention for persons with OA. During the development of this program, the similarities and differences in the treatment of persons with OA and HA were investigated.

## Methods

### Theoretical Model Roadmap

**Figure 1:**
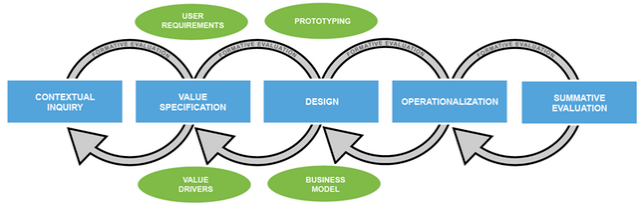
CeHRes roadmap. CeHRes: Center for eHealth Research.

The theoretical Center for eHealth Research (CeHRes) roadmap model was used to develop e-Exercise HA [12]. The CeHRes roadmap is designed for the development and research of eHealth technologies. Cocreation plays a central role in this approach because it anticipates the needs and values of the stakeholders to improve the uptake and the effect of the intervention. Figure 1 shows the 5 stages of the CeHRes roadmap, which consists of connecting formative evaluation cycles. This study focused on the following first 3 stages of the model: contextual inquiry, value specification, and design.

### Stages 1 and 2: Contextual Inquiry and Value Specification

The aim of contextual inquiry and value specification is to establish the most important needs, values, and requirements of the stakeholders in relation to a blended physiotherapy intervention for persons with HA. The input in this step was based on (1) a literature search, (2) experience with the development and implementation of the e-Exercise OA intervention, and (3) focus groups with persons with HA and physiotherapists experienced in the field of hemophilia. These 3 points have been described in detail as follows.

First, a literature search was performed for studies published in PubMed till April 2019 using the search terms “hemophilia” AND “exercise” OR “physiotherapy.” Additionally, we searched for national and international physiotherapy guidelines on hemophilia and OA through patient societies and professional associations and by using search terms “physiotherapy” AND “guidelines and hemophilia” OR “osteoarthritis.”

Second, experience with the development and implementation of the e-Exercise OA intervention was established from previous studies [10,13,14] and by involving the authors of these previous studies in the development process.

Third, 2 focus groups were formed: one group consisted of 5 persons with HA and the other group consisted of 5 physiotherapists experienced in the field of hemophilia. Sessions with the focus groups lasted for around 1 hour and were moderated by MAT. A topic guide was used to discuss the following subjects: the preferred content of physiotherapy for persons with HA, advantages and barriers of usual physiotherapy, information provided and needed, thoughts and beliefs around physical activity (only persons with hemophilia), possible subgroups of persons with HA (only physiotherapists), and advantages and barriers to the use of eHealth. All focus groups were audio recorded and transcribed verbatim. The results were analyzed using a thematic analysis. The data analysis involved reading the transcripts, mapping quotes into codes with the open coding process, and summarizing the codes into themes iteratively.

Thus, information acquired by literature research, experience with a previously developed blended intervention for patients with OA, and focus groups were used to compile the content of the intervention. The authors compiled the first draft of the program. Consequently, all the members of the focus groups individually provided feedback on the draft. Patients were invited to give feedback on the information modules for patients, while physiotherapists were invited to comment on the information modules for physiotherapists. In addition, a hematologist and a social worker commented on the information modules. The physiotherapists collected the feedback with their coworkers in primary care, while patients discussed with their partners or their significant other. The comments were processed by the authors and a second draft was presented to the stakeholders. This process continued until all the members were satisfied.

### Stage 3: Design

During the design phase, the researchers cooperated with “The Health Train,” a commercial eHealth entrepreneur, which was already involved in the previous developed e-Exercise OA app. Currently, e-Exercise interventions are also being developed and studied for low back pain, medically unexplained physical symptoms, and complaints of the neck and shoulder. Knowledge about the design and the functionality of the app studied during the development of other e-Exercise interventions were used for the development of e-Exercise HA. The collaboration with a commercial eHealth entrepreneur can facilitate a long-term implementation of the e-Exercise interventions in physiotherapy care, irrespective of research funding. The possibility of using the same platform for interventions for different patient populations and connecting e-Exercise with the most widely used electronic medical record by physiotherapists in primary care will facilitate the use of e-Exercise by physiotherapists. The research team provided input for the content of the web-based app, and “The Health Train” integrated this content into their platform.

## Results

### Stages 1 and 2: Contextual Inquiry and Value Specification

A recent Cochrane systematic literature review in 2016 [6] provided information on the effectiveness and safety of several exercise protocols for persons with hemophilia, including resistance exercises, isometric exercises, bicycle ergometry, treadmill walking, and hydrotherapy. Most interventions improved one of the following outcomes: pain, range of motion, strength, or walking tolerance. Functional exercises seemed more effective than static exercises for improving strength. No adverse events were reported as a result of any of the interventions. An additional literature search between December 2016 and April 2019 with the search terms “hemophilia” AND “exercise” OR “physiotherapy” yielded 4 original papers that reported similar results after different forms of exercise therapy and manual therapy [15-19]. No guidelines on physiotherapy for persons with HA were available. Instead, parts of the general guidelines for the treatment of hemophilia and parts of the Dutch physiotherapy guidelines for OA were used [1,20]. The demographic characteristics of the participants in both the focus groups are shown in Table 1 and Table 2.

The focus groups provided information on the following themes: current treatment for HA, attitude toward physical activity with HA, information requirements for HA, and values and requirements of e-Exercise for HA.

**Table 1:** Focus Group 1: Demographic characteristics of the participating patients.

Participants with HA^a^	Age (years)	Severity	Hemophilia A/B	Hemophilia joint health score^b^	Hemophilia activity list^c^
Participant 1	56	Severe	A	38	59
Participant 2	54	Severe	A	12	72
Participant 3	67	Severe	A	50	40
Participant 4	40	Severe	A	31	94
Participant 5	76	Severe	A	n.a.^d^	n.a.

^a^HA: hemophilic arthropathy.

^b^High score indicates worse joint status.

^c^High score indicates less limitations in activities, maximum score =100.

^d^n.a.: not assessed.

**Table 2:** Focus Group 2: Demographic characteristics of the participating physiotherapists.

Participants	Overall experience (years)	Experience with hemophilia (years)	Number of persons with hemophilia (n) that were treated	Work setting	Specialization
Physiotherapist 1	30	>10	>10	Primary care	Manual therapy
Physiotherapist 2	26	>10	0-5	Primary care	General
Physiotherapist 3	18	>10	0-5	Primary care	General
Physiotherapist 4	38	>10	>10	Primary care	Manual therapy
Physiotherapist 5	40	>10	>10	Hemophilia treatment center	Manual therapy

#### Current Treatment for Hemophilic Arthropathy

According to the physiotherapists, the basis of the treatment for persons with HA is similar to that of the treatment for patients with OA, and the same physiological principle in OA treatment should be used in HA physiotherapy treatment. The focus group session with the physiotherapists directed at the similarities and the differences between the preferred treatments for OA and HA. This focus group revealed that the treatment for both OA and HA has changed over the last few years from a hands-on approach to a coaching-based therapy, thereby enabling patients to achieve more self-control. The most important difference between OA and HA is that the physiotherapists need to be aware of the symptoms that can indicate a bleeding event while treating a person with HA. Another difference is that persons with HA have more knowledge about their condition—often even more than their primary care physiotherapist. This knowledge plays an important role in their coaching roles. Furthermore, persons with OA have to be motivated to be more active while persons with HA need to learn how to increase the intensity and the duration of the activities and exercises slowly and to avoid peak loads. Patients stated that they benefitted from the exercises and advice on movement behavior as well as the manual therapy. Focus group sessions with persons with HA revealed that the most critical barriers to adhering to physiotherapy were the limited reimbursement by the health insurance, execution of boring exercises, and stubbornness of the patients to accept advise. The patients mentioned that a good relationship with their physiotherapist facilitated their adherence to the physiotherapy treatment.

#### Attitude Toward Physical Activity

Persons with HA explained that they continuously balanced the risk of increasing complaints with the benefits of performing an activity. Patients preferred to do meaningful activities such as walking or cycling in their daily life. They stated that the proud feeling after accomplishing an activity goal motivated them to continue to be active. Some patients mentioned that they preferred to do exercises as well. The important conditions for achieving an activity goal are the choice of the type of activity and tailoring the increase in the intensity and duration with professional guidance. Patients were motivated to perform an activity if they received encouragement but they were demotivated when their complaints were not empathized by others. Patients expressed different opinions about consuming painkillers. Some stated that painkillers enabled them to be active and to relax, while others were afraid that the consumption of painkillers would make them overuse the joint and consequently increase the joint damage.

#### Requirement of Specific Information on Hemophilic Arthropathy

Both persons with HA and physiotherapists in primary care emphasized the need for specific information for physiotherapists in primary care. Physiotherapists in primary care need to know the basic information about hemophilia along with the specific dos and don’ts. Patients did not specify the need for more information for themselves. The physiotherapists stated that basic information about hemophilia need not be provided for such patients. Instead, the patients could benefit from information on the course of their complaints, differences between joint bleeding and arthropathic complaints, how to actively manage HA, and how to cope with the limitations in their daily activities and participation. Short videos were considered as the preferable mode of conveying information to persons with HA and physiotherapists. Information for patients must be provided in a simple language. Physiotherapists were prepared to spend no more than 30 minutes to read or hear all the information that was required. The patients appreciated the communication about their specific situation between the hemophilia treatment center and the primary care physiotherapist.

#### Values and Requirements of Blended Physiotherapy for Persons With Hemophilic Arthropathy

Both physiotherapists and persons with HA believed that an e-Exercise app could add positively to the current physiotherapy treatment for persons with HA. The physiotherapists stated that the frequency of face-to-face visits in this program should be high initially and then decreased during the intervention. The overall number of face-to-face visits should be personalized, which can be determined by the self-management skills and the restraining factors of the patient. The patients stated that the e-Exercise app should include choices for exercises and a variability in the exercises. The patients preferred to set a goal, perform meaningful activities (walking or biking), give feedback on how well they were able to follow the program, and receive pop-ups and an exercise scheme to increase adherence. Physiotherapists stated that they could benefit from a feature in the app through which they could communicate with the patients and be able to consult the hemophilia treatment center for questions. The illustrative quotes for the different themes are presented in Table 3.

**Table 3:** Quotes from physiotherapists and persons with HA^a^.

Theme	Illustrative quote
Current treatment for HA	…*We have our principles of physiology and training. In strength training, overload is needed to make gains for the muscle fibers. This is no different for a person with hemophilia; however, you need to be more careful with increasing intensity.* [Physiotherapist]
Attitude toward physical activity with HA	…*When you have hemophilia, physical activity needs to involve mainly natural movements and things that I just do myself. I try that, I ride my bike, I walk, and I do not take the elevator but the stairs.* [Patient]
Information requirements	…*The difference between patients with OA and those with HA is that because persons with HA have had hemophilia all their life, they have more knowledge about their disease than their 63-year-old neighbor with a painful knee.”* [Physiotherapist]
Values and requirements of e-Exercise	…*So you have complaints and you cannot do certain things. ...the physiotherapist asks what your most important feasible goal is. “…” if you have a problem with your elbow and you cannot put on your sock, it may only be a minimal difference, that can be very inconvenient, and if it is your goal to be able to do that.* [Patient]

^a^HA: hemophilic arthropathy.

### Stage 3: Design

The e-Exercise HA intervention was developed based on the existing e-Exercise OA intervention because of the findings generated from our focus groups, in which the physiotherapists stated that the basis of the treatment for persons with OA and HA is similar. The core components of e-Exercise OA are self-management information, a graded activity approach, and personalized exercises [11]. The need for information modules in e-Exercise HA intervention was expressed by the focus group with physiotherapists as the physiotherapists felt that they lacked the knowledge to educate persons with HA. Therefore, self-management information modules for patients and information modules for physiotherapists were included within e-Exercise HA. Furthermore, focus groups revealed the need for slowly increasing an activity and avoiding peak loads, goal-setting and performing meaningful daily activities. Those needs were met by the graded activity approach. Personalized exercises were suggested by persons with HA and have been reported in a previous study [6]. Feedback from the stakeholders on the drafts mainly consisted of changes to the content of the information modules and the script of the information videos. Since the web-based environment was already developed in cocreation with the end users and adjusted based on the experiences during the trial, the design of the web-based environment was not changed.

### e-Exercise Hemophilic Arthropathy

This 12-week intervention integrates face-to-face physiotherapy with a smartphone app. Physiotherapists can log into the website, which offers the platform with the physiotherapist-specific information about HA. The physiotherapists can create an account for each patient. In this account, physiotherapists can create and personalize an exercise program for each patient and monitor the progress. The website allows physiotherapists to continuously adapt the treatment program according to the needs of the individual patient. Patients can log into their personal account in a smartphone app. In this app, the patients receive weekly modules with information and are able to review their personal physical activity program and their personalized exercises. They can give feedback on the performance of the prescribed program and receive tailored feedback messages. The components of e-Exercise HA have been further explained below. A screenshot of the e-Exercise HA app is shown in Multimedia Appendix 1.

#### Self-Management Information Modules

The information modules consist of text blocks supported by short videos [1,6,20-24]. For the physiotherapists, the following 5 information modules are available: (1) What is hemophilia? (2) Development of HA. (3) HA and physiotherapy. (4) Identifying a bleed. (5) Organization of care. The patients receive the following information modules on their smartphone app every week: (1) What is HA? (2) Pain and HA. (3) Joint bleed or HA? (4) Physical activity and HA. (5) Responsible exercise. (6) Arthropathy management. (7) A physical activity plan. (8) Maintaining an active life. (9) Maintaining healthy body weight. (10) Dealing with the surrounding people. (11) Fatigue. (12) What can you do? After every module, patients are invited to answer a reflective question in that particular theme. Physiotherapists are encouraged to further discuss the themes during the next face-to-face visit. When the patients feel that a specific theme does not suit their personal situation, they can choose to skip that specific information module.

#### Graded Activity

The behavioral graded activity approach is directed at increasing the amount of physical activity in a time-contingent way so that the activities are gradually increased by present quotas [25]. First, the persons with HA select their most problematic activity (eg, walking). The initial baseline measures, in which the patients perform the selected activity at home to the limit of tolerance (pain-contingent), are then performed for a week and will be registered in the app. Second, after these baseline measurements are recorded, the patients set their individual treatment goal for the selected activity. During this procedure of goal setting, the physiotherapist acts as a coach only, as it is essential that the goal is the patient’s own internal goal. Throughout the program, this activity has to be performed 3 times a week for 11 weeks. The duration will be gradually increased in a time-contingent manner to reach the final goal at the end of the program. The weekly program will be automatically generated starting at 50% of the baseline assessment. On self-chosen moments (eg, every Monday at 8 PM), patients receive reminders on their smartphone to perform this activity. Patients can turn the timer on and off when they perform their activities so that the physiotherapist can monitor the progression. Third, tailored feedback messages are provided at the end of the activities with the aim of motivating or slowing down the patients with their program by using the principles of operant conditioning. The progression will be evaluated during the face-to-face visits.

#### Exercises

e-Exercise HA is linked to a database of over 1500 exercises, which are supported with texts and videos. In the face-to-face sessions, physiotherapists can start an exercise program by choosing personalized exercises and parameters. On their apps, patients can recall their exercises, including the parameters and the information about their performance. Similar to that in the graded activity approach, the physiotherapist is able to monitor the performance of the exercises. During the face-to-face visits, the performance of the exercises can be evaluated and adjusted when needed.

#### Face-to-Face Sessions

Face-to-face sessions are conducted to recall web-based self-management modules and to discuss how the themes affect the patient’s personal situation by evaluating the answers on the reflective questions. The progress in the graded activity program and in the performance of the exercises can be visualized in the management portal of the website used by the physiotherapist. When needed, the graded activity program and the prescribed exercises can be adjusted. We propose 6 face-to-face visits during the 12-week program, with a decreasing frequency as the program progresses. The visits will be proposed to be scheduled on weeks 1, 2, 4, 6, 9, and 12. However, the number of visits and the timing of the visits depend on the personal needs of the patients, including the self-management skills and digital skills.

## Discussion

### Principal Findings

In this study, we developed a blended physiotherapy intervention for persons with HA, which meets the needs of the persons with HA and the physiotherapists in primary care. To enable successful implementation, the intervention was developed by cocreation with stakeholders such as persons with HA, physiotherapists, software developers, and researchers. Our focus group study with persons with HA and physiotherapists yielded the following findings: physiotherapy for persons with HA is similar to that for persons with OA, specific tailored information on rare diseases such as hemophilia and HA is required for patients and physiotherapists, patients prefer meaningful daily life activities and want to work toward achieving a goal, patients prefer choices and variability in exercises, and the exercise program needs to be personalized. Both physiotherapists and persons with HA agreed that integration of an e-Exercise app within physiotherapy treatment could be beneficial for the treatment of HA. The most important difference between providing treatment for OA and HA is that physiotherapists need to be aware of the disease symptoms that can be indicative of bleeding when treating a person with HA.

### Comparison With Other Studies

Currently, several eHealth apps are being developed and used in hemophilia care [26]. However, these are mainly videoconferencing apps and apps the report bleeding and the use of clotting factor replacement therapy. No study has yet described smartphone apps that support physiotherapy in persons with hemophilia or related bleeding disorders [26]. Focus groups in this study showed that the Dutch physiotherapists and persons with HA showed a positive attitude toward the use of a blended physiotherapy intervention. This is in accordance with previous studies showing that blended eHealth intervention is feasible and effective in persons with OA, although some physiotherapists expected to face some barriers in this intervention mode [11,14,27]. An important barrier for physiotherapists to use e-Exercise OA was that it could be used for only 1 disorder. The current expanding of the platform with e-Exercise programs for several disorders is expected to facilitate the use of e-Exercise HA [28]. Another barrier was that some physiotherapists felt that the program interfered with their professional autonomy. However, given the limited knowledge of primary care physiotherapists about hemophilia, it is expected that this is less of a problem for e-Exercise HA. In accordance with previous studies, the requirement of e-Exercise determined in this study included a smartphone app with information for patients, goal-setting features, and a personalized exercise program [10]. The need for information modules for physiotherapists is specific for hemophilia, which is understandable, given the rare nature of the disease.

### Strengths and Limitations of This Study

Hampered uptake and implementation of eHealth solutions in clinical practice is a common problem [29]. A strength of this study is that the intervention was developed in cocreation with the end users (ie, patients and physiotherapists) according to the CeHRes roadmap in order to accelerate implementation at a later stage. Adding information modules particularly for physiotherapists was a strong recommendation by the stakeholders and is expected to improve the quality of care. A limitation of this study is that only Dutch stakeholders were invited. We expect that different barriers and facilitators might be present in different countries, thereby limiting the generalizability of our findings in different countries. Moreover, only patients aged over 40 years participated in the process of cocreation. This limits the generalizability of the findings to adolescents and young adults. Furthermore, the feasibility and the effectiveness of this intervention have not yet been investigated and will be investigated at a later stage of this project. The intervention in this study was built upon an existing intervention for persons with OA and is part of a platform for blended physiotherapy interventions for several disorders. This has enabled us to learn from the results of these interventions and will lower the threshold for the implementation of our intervention for persons with a rare disease. The use of an existing platform also limited us to make major changes to the web-based environment. This intervention has been developed in cocreation with stakeholders as well and we expect that preferences regarding this topic will not be very different from those of our population. The cocreation process in this study focused mainly on the content of the intervention and less on the web-based interface.

### Clinical Implications and Future Research

A possible use of e-Exercise HA is by physiotherapists who are associated with a hemophilia treatment center and who are able to treat patients who visit the clinic a few times, but not on a weekly basis. Moreover, e-Exercise HA can support physiotherapists in primary care, who have no experience with hemophilia but can treat persons with HA locally. We expect blended physiotherapy to lead to less face-to-face visits. This could increase the accessibility of physiotherapy for persons with HA, leading to lower costs and more flexibility for patients. Furthermore, we expect that e-Exercise HA will improve self-management with respect to the movement behavior because patients are provided with information modules and are stimulated to perform activities at home. Future research should be directed toward investigating the effectiveness of this intervention and the implementation of this intervention in the Netherlands. To enable the international implementation of this program, the barriers and the facilitators of the stakeholders in different countries need to be investigated.

### Conclusion

We developed a 12-week blended physiotherapy intervention that integrates face-to-face physiotherapy sessions with a smartphone app for persons with HA. This personalized intervention consists of self-management information, a graded self-chosen activity program, and video-supported personalized exercises, which integrates face-to-face sessions and a smartphone app.

## Appendix

Multimedia Appendix 1 Screenshot of an information module for patients.

## Abbreviations

CeHRes: Center for eHealth Research
eHealth: electronic health
HA: hemophilic arthropathy
OA: osteoarthritis
