# Supplementary figures and images for: A Blended Physiotherapy Intervention for Persons With Hemophilic Arthropathy: Development Study
Source: J Med Internet Res. 2020 Jun 19;22(6):e16631. doi: 10.2196/16631 (PMC7334756; doi:10.2196/16631)

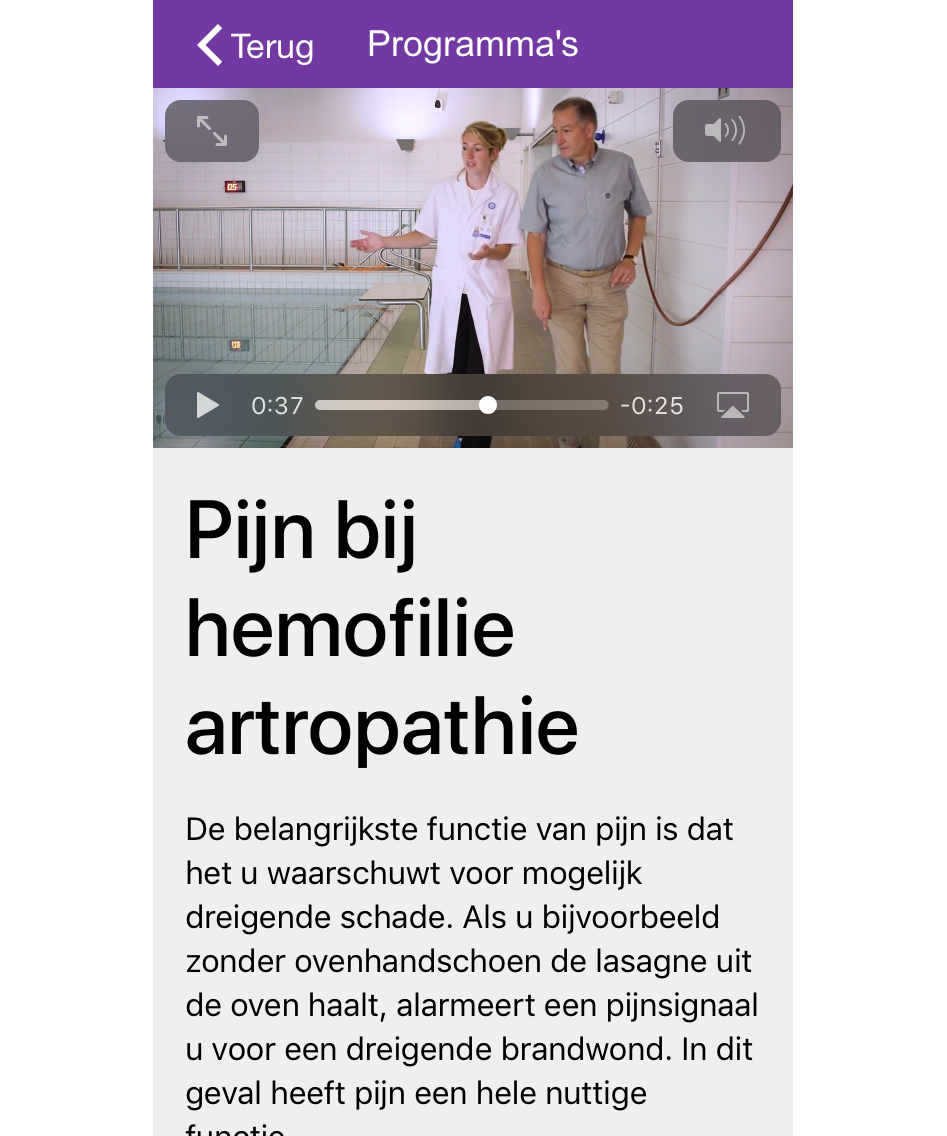

Supplement: Multimedia Appendix 1 [file jmir_v22i6e16631_app1.png]
